# Supplementary material for: Are carbon emissions trading and green financial instruments synergistic? -Comprehensive quantitative research based on content analysis
Source: PLoS One. 2024 Mar 7;19(3):e0298601. doi: 10.1371/journal.pone.0298601 (PMC10919601; doi:10.1371/journal.pone.0298601)
Supplement: S1 Data — (ZIP) [file pone.0298601.s001.zip › Data/地级市Z_F工作报告.zip!/╔┬╬≈/╔╠┬σ╩╨/└┤╫╘╣1⁄2╓┌║┼í╛┬φ┐╦╩2╛▌═°í┐_2012.docx]

各位代表：

现在，我代表第二届市人民政府向大会作工作报告，请予审议，并请市政协委员和其他列席人员提出意见。

一、过去五年工作回顾

市二届人大一次会议以来，在市委的正确领导下，市政府团结依靠全市人民，坚持以邓小平理论和“三个代表”重要思想为指导，深入贯彻落实科学发展观，紧紧抓住国家深入推进西部大开发、实施《关中—天水经济区发展规划》和扩大内需等一系列重大历史机遇，励精图治，积极进取，全面完成了市二届人大一次会议确定的各项目标任务，全市经济社会保持了持续快速发展的势头。

(一)综合经济实力显著增强。五年来，我们紧扣发展第一要务，坚持不懈扩内需、保增长、扩总量，主要经济指标连续保持两位数增长。2011年实现生产总值362.88亿元，增长15.1%，是2006年的2.6倍，年均增长14.5%。财政总收入26.75亿元，增长25.9%，是2006年的3.7倍，年均增长29.8%。地方财政收入16.5亿元，增长37.3%，是2006年的4.9倍，年均增长37.4%。全社会固定资产投资308.36亿元，增长30.9%，是2006年的3.1倍，年均增长25.3%，五年累计完成1116.61亿元，是前五年的3.2倍。全社会消费品零售总额92.18亿元，增长16.4%，是2006年的2.3倍，年均增长18.2%。城镇居民人均可支配收入17344元，增长17.1%，较2006年净增9573元，年均增长18.2%；农民人均纯收入4586元，增长27.2%，较2006年净增2977元，年均增长22.4%。金融机构各项存款余额446.59亿元、贷款余额187.48亿元，较2006年增加279.64亿元和114.96亿元，年均分别增长21.7%和20.9%。

(二)特色经济进一步发展壮大。五年来，我们围绕做大做强特色产业，坚持不懈转方式、调结构、促转型，优势主导产业支撑效应显著提升。工业转型升级步伐加快，2011年全市规模以上企业120户，其中产值过亿元企业30户，较2006年增加29户。实现全部工业总产值306.79亿元、增加值96.31亿元，分别增长48%和25%，是2006年的4.5倍和4.7倍。现代材料、生物医药、绿色食品产业产值占到工业总产值的80.3%，较2006年增加11.6个百分点。商丹园区被列为省级园区，7个县域工业集中区被列为省级重点，入园企业达到188户，规划的10条循环经济产业链8条启动建设，其中5条基本建成，20个循环经济标志性工程在建16个，11个资源综合利用示范重点项目已经建成，五年淘汰落后产能211.2万吨，单位生产总值能耗降低19.6%。农业现代化水平不断提高，核桃、板栗、药材、茶叶、畜牧等特色产业快速发展，建设省级现代农业示范园4个，认定省级重点龙头企业12家，农业特色产业收入占到农业总产值的63%，较2006年提高17个百分点，2011年粮食总产59.8万吨。现代服务业快速发展，建成4A景区1个、3A景区2个，成功举办了两届秦岭生态旅游节，“秦岭最美是商洛”唱响全国，2011年接待游客1700万人次、收入68亿元，分别是2006年的15倍和28倍。肯德基、华润万家、国美电器和苏宁电器等23家大型连锁企业落户商洛，物流配送中心发展到23家，城乡大中型超市发展到13家，连锁化农家店覆盖所有镇办和85%的行政村。

(三)基础设施和生态环境进一步优化。五年来，我们全力推动可持续发展，坚持不懈抓项目、破瓶颈、增后劲，发展条件显著改善。累计实施市级重点项目481个，完成投资600亿元。以交通为重点的基础设施建设不断加强，西康铁路复线开工建设，沪陕、包茂、福银三条高速商洛段建成通车，西商二线和榆商高速洛南段即将建成，高速公路通车里程达到351公里，改造建设国省道、通乡油路、县乡公路2700多公里、通村水泥路6500多公里、通达工程2600多公里。电力保障能力明显提高，建成了商南鹿城330千伏高压输变电和一批城乡电网改造工程。农村基础设施条件持续改善，实施农业综合开发项目63个，建设新农村示范村270个、扶贫开发重点村707个，实施“四改三建”6720户，新修“四田”29.6万亩，综合治理小流域146条，治理水土流失3399平方公里，解决了105万人的饮水困难。生态环境保护力度加大，完成重点工程造林173.5万亩，实施重点区域绿化30万亩，“十一五”时期森林覆盖率由52.4%提高到62.3%。市污水、垃圾和医疗固体废物处理厂建成投运，六县污水处理厂全部建成试运行，超额完成了省上下达的主要污染物减排任务，2011年市区空气质量达到或好于国家二级标准的天数为351天。

(四)城镇化进程快速推进。五年来，我们坚持城乡统筹发展，坚持不懈抓规划、兴建设、强管理，城乡面貌发生巨大变化。完成了中心城市和六县县城总体规划编制，制定了商丹一体化建设方案，城市控制性规划覆盖率达到80%，比2006年提高45个百分点。中心城市建设累计投资30多亿元，相继完成江滨大道、丹江公园和体育馆等46个市区重点工程，环城北路、丹南新区路网等重点项目快速推进，市区建成区面积25平方公里，比2006年增加7平方公里。城市绿化工作不断加强，市区绿化覆盖率达到40%，五年提高6个百分点。六县县城、沙河子省级重点示范镇和7个省级重点镇建设进度加快，实施村改居项目74个，11.1万农村居民进城落户，全市城镇化率达到40.2%，五年提高16.4个百分点。中心城市被命名为省级卫生城市和园林城市，创建省级环保模范城市有序推进，柞水县城被命名为国家卫生县城和全国文明县城，镇安、洛南、商南三县县城被命名为省级卫生县城，丹凤县创建省级卫生县城通过省上验收。

(五)改革开放取得重大突破。五年来，我们深入推进改革开放，坚持不懈抓招商、促创新、增活力，开放开发水平显著提高。医药卫生体制改革取得重大进展，镇安农村医改在全国评估中名列第一，药品“三统一”覆盖所有基层医疗单位。文化体制改革加快推进，市直文化单位改革基本到位。事业单位人事和分配制度改革任务完成，市、县区和乡镇机构改革全面到位。投融资和金融体制改革深入推进，成立了城投公司、创投公司、市中小企业担保公司和秦宇矿业公司，设立小额贷款公司9家、融资性担保公司4家。国企改革、国有资产管理体制改革以及集体林权、土地流转、粮食流通、种子管理、基层供销社、水务体制和农牧综合服务体系改革稳步推进。保险覆盖范围大幅拓展，农村小额人身保险、烤烟保险、设施蔬菜大棚综合保险、农村房屋保险和核桃产业保险等业务相继开展，累计实现保费收入32.9亿元。主动承接东部产业转移，深入开展招商引资，成功引进比亚迪、中金、国电、华电、延长等19家国内外500强企业，马来西亚客商在我市设立了全省首家外商独资投资性公司，五年招商引资到位资金377.9亿元，是前五年的14倍。认真落实促进全民创业的政策措施，私营企业、个体经营户发展到4362家、8.9万户，较2006年分别增加2432家和2.1万户，非公有制经济增加值占全市生产总值的比重达到49%。

(六)和谐社会建设成效显著。五年来，我们竭力保障和改善民生，坚持不懈办实事、解民忧、促和谐，人民群众幸福指数明显提高。坚持将新增财力的80%以上用于民生，累计实施民生项目1.37万个，完成投资147亿元。转移农村富余劳动力281.8万人次，新增城镇就业岗位5.6万个，城镇登记失业率控制在4%以内。城乡居民养老保险实现制度全覆盖，最低生活保障实现应保尽保，五保对象集中供养率达到58.6%，高龄老人生活补贴政策全面落实，5.35万农村贫困残疾人享受定期定额生活补贴。建成各类保障性住房4.4万套，改造农村危旧房3.1万户，实施移民搬迁1.67万户6.78万人，建设集中安置点213个，42万贫困人口解决温饱并实现稳定脱贫。全面发展各项社会事业，教育办学条件不断改善，教学质量稳步提高，高考二本以上上线率连续四年全省第一，“蛋奶工程”惠及16.6万寄宿制中小学生和农村幼儿。四级医疗服务体系进一步健全，群众看病就医条件极大改善，新农合参合率98.99%，连续八年全省第一。自主创新能力不断增强，启动建设了市材料科学工程院、陕西循环经济工程研究院商洛分院、商洛尾矿资源综合利用研究院等科技创新平台，建立市级企业技术中心8家，申请专利413件，授权专利173件，鉴定科技新成果20项。文化和广电事业快速发展，建立了市县镇村四级文化服务体系，广播电视覆盖率达到93.6%和98.7%。体育基础设施不断改善，全民健身活动日趋丰富，成功举办了全国竞走大赛和国际青年女篮四国挑战赛。人口和计划生育工作持续加强，出生人口政策符合率保持在95%以上，人口增长连续19年低于全省平均水平。国土资源管理和用地保障工作全面加强，矿产资源保护开发和整顿受到国土部充分肯定。社会管理创新工作扎实推进，在全省率先组建了综合应急救援队伍，应急处置能力进一步增强。“平安创建”活动深入开展，建立了县区信访联合接待大厅，公安“三基”工作得到加强，安全生产形势总体平稳，人民群众对社会治安的满意率连续五年位居全省前列。

在推进经济发展和构建社会和谐的实践中，统计、工商、质监、人防、气象、水文、方志、宗教、外事、侨务、老龄、档案、慈善、工商联、红十字会、妇女儿童、国防动员等工作都取得了新的成绩。

五年来，我们把依法行政放在重要位置，坚持市政府领导集体学法制度，自觉接受人大的法律监督和政协的民主监督，认真办理人大代表建议和政协委员提案。主动加强与人民群众的联系，积极办理回复网民留言和各类反映，重视听取各方面的意见和建议。加强行政监察、审计监督和责任考核，公务人员的廉政勤政意识进一步增强。扎实推进机关效能建设，完善了抓落实工作机制，提高了政府工作效率。

各位代表，五年来，我们经受了诸多严峻考验和挑战，各项工作都取得了显著成绩，谱写了商洛发展史上新的重要篇章。这一系列深刻变化，极大地增强了全市人民的光荣感和自豪感，极大地坚定了全市人民加快发展的信心和决心，极大地激发了全市人民建设幸福家园的积极性和创造性。今天的商洛，正以蓬勃的朝气与活力向着富民强市的宏伟目标阔步前进！

各位代表，五年的发展令人鼓舞，五年的成就来之不易，这得益于省委、省政府和市委的正确领导，得益于市人大、市政协的监督支持，得益于历届政府打下的坚实基础，更得益于全市人民的团结奋斗和顽强拼搏。在此，我代表市人民政府，向全市广大干部群众，向各级人大代表、政协委员、各民主党派、工商联、人民团体、离退休老同志，向驻商部队指战员、武警官兵和公安干警，向所有关心、支持和参与商洛改革发展的各界人士以及商洛籍在外父老乡亲，表示衷心的感谢并致以崇高的敬意！

在肯定成绩的同时，我们也清醒地看到，全市经济社会发展中仍然存在着不少困难和问题：经济总量小，产业规模小，非公有制经济发展不快；经济增长方式粗放，节能减排任务艰巨；观念和体制性束缚依然存在，市场化和对外开放水平不高；公共服务能力不强，保障和改善民生的任务依然艰巨；官僚主义、形式主义和奢侈浪费现象仍不同程度存在，发展环境还有待进一步优化。对于这些问题，我们必须高度重视，采取得力措施，认真加以解决。

二、今后五年政府工作的总体要求和奋斗目标

按照省委、省政府和市第三次党代会的部署，今后五年政府工作的总体要求是：高举中国特色社会主义伟大旗帜，以邓小平理论和“三个代表”重要思想为指导，深入贯彻落实科学发展观，进一步解放思想、更新观念，深化改革、扩大开放，以科学发展、富民强市为主题，以发展循环经济为主线，以保障和改善民生为出发点和落脚点，继续坚持“三大战略”、“四大带动”，切实转变发展方式，不断优化经济结构，着力提高发展质量，全力打造“三区四基地”，努力建设绿色现代、开放和谐、文明宜居的幸福商洛。

今后五年的奋斗目标是：

经济综合实力进一步提升。经济增长高于全省平均水平，主要经济指标翻番增长，2016年全市生产总值达到750亿元，是2011年的2.1倍，财政总收入达到60亿元，是2011年的2.3倍。

基础设施条件进一步改善。全社会固定资产投资五年累计达到2400亿元，是前五年的2.2倍，公路交通、城乡面貌和生态环境进一步优化，全市城镇化率提高到50%，森林覆盖率达到66%。

人民生活水平进一步提高。2016年城镇居民人均可支配收入36000元，是2011年的2.1倍，农民人均纯收入达到9500元，是2011年的2.1倍，人民生活更加殷实，幸福感进一步增强。

实现上述奋斗目标，我们必须以科学发展观统领全局，在推进工作中切实把握好以下四点：

一是坚持发展为先，构筑科学发展、加快发展新格局。今后五年，我们将坚定不移地继续实施项目带动战略，扎实抓好高速公路、铁路复线、国省道改造、产业路和农村公路建设，积极争取西安商洛机场和大秦岭通用航空机场项目启动建设，构建现代化综合交通网络。坚定不移地推进新型工业化，以打造“四大基地”为目标，以实施“1358”工程为重点，大力发展新材料、节能环保等战略性新兴产业，构建现代优势特色产业体系。坚定不移地推进农业现代化，以“一村一品”、“一县一产业”为抓手，大力发展现代农业、高效农业，建成一批农业优势产业带和现代农业产业园。坚定不移地推进新型城镇化，打造关天经济区次核心城市，按照“北上、南扩、东进、西优”的思路，加快中心城市建设步伐，全力推进商丹一体化，加强县城和重点镇建设，构建特色鲜明的现代城镇体系。

二是坚持生态为重，形成循环发展、持续发展新优势。今后五年，我们将坚持生态立市战略不动摇，加快实施秦岭山地生态功能区和水源保障区生态环境综合保护工程，大力实施丹江流域综合治理，全面绿化城周及公路沿线荒山，积极创建国家园林城市、生态县区、生态镇和生态村，进一步强化节能减排目标责任考核，加强重点行业、重点区域环境整治，抓好农村环境综合治理，让商洛的山更绿、水更清、天更蓝。加快推进全国循环经济示范区和尾矿综合利用示范基地建设，重点抓好10大循环产业链、20个循环经济标志性工程建设，培育1个国家级10个省级50个市级循环经济示范企业，建成3个省级5个市级循环经济园区，实现创建全国循环经济发展示范城市目标。

三是坚持创新为魂，注入开放发展、创新发展新动力。今后五年，我们将继续推进经济、社会和行政管理体制改革，深入推进企业、财税和投融资等领域改革，构建充满活力、富有效率、更加开放、科学发展的体制机制。全面加强科技和人才工作，大力实施“51158”工程，提升科技创新能力，加快科技成果转化，使创新成为我市发展最强劲的动力。全方位扩大对外开放，加快发展开放型经济，提高经济的外向度和融合度。全面实施中小企业培育计划，大力倡导全民创业，鼓励自主创业，支持家庭创业，不断提升非公有制经济发展水平。

四是坚持民生为本，谱写和谐发展、安全发展新篇章。今后五年，我们将顺应人民群众的新期待，健全新增财力80%用于民生的长效机制，提升基本公共服务水平，提高全市人民群众的生活品质和幸福指数。加强和创新社会管理，健全群众利益维护机制，提高社会管理科学化水平。始终把保护人民生命财产安全放在首位，构建完善公共安全和防灾减灾体系，巩固发展社会安定、百姓安宁的良好局面。

三、努力做好今年的各项工作

今年党的十八大将要召开，也是新一届政府的开局之年。做好今年各项工作，责任重大、意义深远。全市经济社会发展的预期目标是：经济增长13.5%左右，财政总收入和地方财政收入均增长19%，城镇居民可支配收入和农民人均纯收入分别增长15%和16%，城镇登记失业率控制在4.5%以内，CPI涨幅控制在4%左右，人口自然增长率控制在6.5‰以内，节能减排指标控制在省上规定的范围之内。

(一)进一步强化项目带动，增强经济社会发展后劲

坚持项目带动战略不动摇，狠抓重大项目建设不放松，继续保持投资稳定增长。全市安排1亿元以上项目前期费，重点推进150个重大项目前期工作。实施市级重点项目150个，年度投资220亿元，确保全社会固定资产投资增长不低于26%。

一是强力推进产业项目建设。实施重点产业项目100个，年度投资110亿元。重点抓好国电公司丹江商南境内梯级水电开发、凯迪公司生物质能源综合开发和同兴轧钢公司40万吨中板生产线等项目建设，启动热电厂、汽车零部件、镇安抽水蓄能电站等项目建设，加快天成万豪、万国生态度假等五星级酒店建设，力促丹凤尧柏龙桥废弃物综合利用、西部鑫兴钼深加工等项目建成投产。

二是抓好重大基础设施建设。积极配合西康、西合铁路复线建设，争取西商二线“五一”前通车、山柞高速开工建设。积极推进202、307省道洛南过境路、102省道镇安过境路建设，加快建设商郧路等出省通道，改造县乡公路125公里，建设通村水泥路200公里。加快市区一级客运站建设，镇安、柞水两个二级客运站建成投运。实施330KV旬商线和330KV丰张输变电工程，新建改造110KV输变电线路4条、35KV线路4条，完成392个行政村农网改造任务。扎实推进无线城市建设，新建城乡基站350个。

三是强化项目建设保障和监管。不断拓宽项目融资渠道，加强政府投资引领作用，积极向上争取投资，扩大信贷投放规模，鼓励引导民间投资，力争城投债顺利发行。强化立项、审批、用地、征迁等关键环节的服务保障，加强工程监理、监察和审计，对所有政府性投资项目严格预决算审查，确保工程质量、资金安全和投资效益。

(二)加快转变经济发展方式，推动产业结构优化升级

进一步强化循环发展理念，大力发展战略性新兴产业，加快现有产业优化升级步伐，不断增强经济发展的协调性和竞争力。

一是深入推进循环经济发展。认真落实《陕西省循环经济促进条例》，积极创建国家级循环经济示范城市，继续抓好山阳丰源公司3000吨氮化钒扩建等循环经济标志性工程，抓好丹凤磁铁尾矿综合利用和商南华瑞公司微晶玻璃等14个尾矿资源综合利用示范项目建设。认真落实《加快推进循环发展的实施意见》，市本级每年安排不低于5000万元专项资金用于支持循环经济发展。

二是加快新型工业化进程。全面落实增值税转型改革政策，对纳入省上的20户重点企业，将新增增值税地方留成部分的50%用于支持企业发展。继续落实“一企一策”包抓措施，引导企业强化内部管理，降低生产成本，开拓销售市场，扩产提质增效，全年规模以上工业增加值增长26%以上。加快园区基础设施和标准化厂房建设，完善仓储物流、创业基地和技术服务中心等配套体系，注重引进关联配套企业，新增入园企业40家。市上安排1000万元资金用于科技创新和产业发展，落实支持企业建立研发中心和增加研发投入的各项政策，支持科研院所、大专院校以技术入股等形式与企业合作。实施企业家培训工程，推动企业管理升级，提升企业软实力。

三是大力发展现代服务业。持续加大景区景点建设力度，力争金丝峡5A级景区通过国家验收，至少创建两个4A级景区。抓好市县区旅游接待服务中心建设，加快建设凤凰古镇、漫川古镇等5个特色旅游名镇，不断拓展乡村旅游市场。加大旅游宣传促销力度，精心筹办第三届秦岭生态旅游节和茶叶节，确保接待游客突破2000万人次，实现综合收入90亿元。制定出台促进文化产业发展的实施意见，重点支持文化创意、广告演艺、休闲养生和会展节会等产业，推动文化与旅游的深度融合。积极鼓励发展社会化养老、家政和医疗保健等服务业。围绕打造区域物流中心，抓好商山物流园和农产品冷链物流建设，完善物流和检测检验等公共平台。加快完善银行业组织体系，推进国有商业银行恢复县区经营机构，引进股份制商业银行在我市设立分支机构，支持长安银行商洛分行尽快开业运营。改善金融生态环境，发挥担保公司作用，不断拓展保险业务，全年新增贷款40亿元，实现保费收入10亿元，力争服务业增加值增长15%以上。

四是千方百计扩大城乡消费。把提高城乡居民收入摆在首位，完善促进居民消费的政策，提升居民消费能力。在落实农民原有增收措施的同时，更加注重增加经营性和财产性收入。全面加强劳动监察工作，督促企业严格执行工资指导线制度、最低工资标准和各项保障政策，切实解决农民工工资拖欠问题。严格落实中省有关政策，继续提高企业退休人员养老金水平。认真落实农机具下乡政策，积极参加各类大型节庆及商品展销活动，加快培育电子商务、休闲、健身等消费热点。加快大型商贸市场建设，确保商洛晨光城和通江国际电子数码城投入运营。积极推进“农超对接”、“农社对接”、“农餐对接”，继续抓好县级农贸市场升级改造和社区标准化蔬菜市场建设，改造和新建配送中心3个，新建各类连锁经营网点300个，升级改造农贸市场4个。继续加强市场秩序整顿，严厉打击制售假冒伪劣商品行为，进一步改善城乡消费环境，全社会消费品零售总额增长18%。

(三)全面加强“三农”工作，加快现代农业发展步伐

以新农村建设为抓手，加强农业农村基础设施建设，大力发展现代农业，促进农业和农村经济持续繁荣。

一是认真落实强农惠农富农政策。不断增加对“三农”的投入，按时足额兑现粮食、生猪、农资、良种、农机具购置等各项农业补贴，切实保护和充分调动广大农民的生产积极性。大力推广高效增产技术，提高粮食单产，确保粮食总产55万吨左右。

二是积极推进规模化经营。坚持依法、自愿、有偿的原则，鼓励引导农户以转包、出租、互换、转让等方式，有序推进土地承包经营权流转，吸引资本、技术等要素加速聚集。加快现代农业产业基地建设，重点抓好核桃、马铃薯、设施蔬菜、中药材等四大优势产业，加快发展生猪、板栗、茶叶、烤烟等区域特色产业，建成省市现代农业示范园7个，建设马铃薯基地60万亩，发展设施蔬菜7000亩、烤烟12万亩、中药材10万亩，新发展核桃、板栗基地30万亩，建设冷水鱼和大鲵驯养繁殖基地20个，新建标准化规模畜禽养殖场100个，肉、蛋、奶产量分别达到20万吨、6万吨和1700吨。

三是提高农业产业化水平。积极引进省内外有实力的龙头企业，推动市内龙头企业整合重组，新发展市级龙头企业10个、省级10个、国家级1个。加快推进农业科技进步，完善农村科技服务体系，培育壮大农民专业合作社，提高农业生产的组织化水平。积极开展名牌产品和著名商标创建工作，年内认证“三品”15个，注册农产品商标15个。

四是全面加强农田水利建设。深入开展中小流域综合治理，完成“丹治”二期首批19条小流域建设任务，新修加固堤防200公里，除险加固病险水库7座，治理水土流失800平方公里，建设基本农田7万亩，修建“四田”4.5万亩，改造提高3万亩，增加灌溉面积2.5万亩。积极实施农村饮水工程，解决20万人饮水困难。持续开展农业综合开发，治理土地3.3万亩。加强中小河流水文监测站点建设，启动建设气象数据楼，多普勒天气雷达投入使用。

五是扎实推进扶贫开发工作。深入贯彻落实中央新十年扶贫开发纲要，按照中省扶贫标准确定工作到村、扶贫到户的范围和对象，制定落实相关政策和措施，启动建设72个新农村和35个扶贫整村推进项目，建立产业扶贫示范村30个，实现6万低收入人口稳定脱贫。

(四)加强生态环境建设，提高两型社会建设水平

加快建设资源节约型和环境友好型社会，完善节能减排政策引导和市场机制，大力实施重点生态建设工程，走生产发展、生活富裕、生态良好的文明发展道路。

一是严格控制污染物排放。坚决落实节能减排目标责任制，建立完善监测预警和评价体系，突出抓好小岭铁矿区、黄龙钼矿区、中村钒矿区、商南县钒业开发小区和商丹园区等重点区域环境整治，严禁超标排放，严厉打击环境违法行为。加快各县污水处理管网建设，上半年污水处理厂必须正常运行。抓好各县垃圾处理厂和镇办垃圾、污水处理设施建设，有条件的地方要延伸到村。加强烟煤禁烧工作，进一步优化空气质量。

二是继续加强节能降耗工作。严格执行固定资产投资节能评估暂行办法，全面推进工业、交通和公共机构节能，年内淘汰所有机立窑水泥生产线。进一步加强建筑节能，鼓励使用太阳能和其他可再生资源，逐步改造现有城市耗能灯具，新上亮化工程一律采用节能灯，全面提升城市节能水平。

三是不断加强生态环境建设。深入推进省级环保模范城市创建工作，启动农村环保重点村建设试点，完成商南、镇安、柞水省级生态县创建任务。扎实开展退耕还林、天然林保护和长江防护林工程建设，继续抓好城市和城周绿化工作，全年完成造林绿化30万亩，千里绿色长廊25.6万亩，义务植树800万株，千河固堤绿化1000公里。加强丹江源头生态保护，切实抓好河道采砂管理，加快实施地质灾害治理工程，开展重点矿区地质环境恢复和尾矿库专项整治。

四是进一步加大资源节约力度。加强城镇供水管网改造，完善节约用水激励政策，万元GDP水耗下降5%。把尾矿综合利用作为矿产项目准入的前置条件，提高矿产资源循环开发和节约利用水平。建立健全节约集约用地考核制度，深入推进土地整治和“空心村”治理。

(五)加大城镇建设力度，加快城乡统筹发展

围绕建设文明宜居城市，开展“城市建设管理年”活动，进一步强化城镇规划、建设和管理，全面改善城镇人居环境，全市城镇化率提高到42%。

一是加快推进商丹一体化建设。编制完成商丹一体化建设规划，启动丹凤县撤县设区工作，抓紧实施312国道二改一工程，积极做好丹江干流综合治理前期工作，力争早日列入中省盘子并开工建设。

二是加大中心城市建设力度。加快编制中心城市和重要区域控制性详细规划，启动编制城市道路、城市景观、集中供热供气等专项规划，中心城市详细规划覆盖率达到90%。坚持新城建设与旧城改造并举，西街旧城改造和莲湖东侧旧城改造完成拆迁和安置房建设，加快推进环城北路、商鞅大道等重点工程建设，完成龟山大道、西商二线市区连接线跨丹江大桥、市建筑垃圾处理场和城区雨污分流管网等工程，启动建设文化艺术中心，积极推进城中村改造。加快城市人防工程建设，合理开发利用地下空间。继续抓好城区“退二进三”工作。

三是加快县城和重点镇建设。突出县域历史文化特色，合理确定县城规模、建筑风格、功能定位和人口密度，统筹安排县城空间发展次序，完善落实相关政策措施，加快县城基础设施建设，不断提高县城综合承载能力。全面推进沙河子省级重点示范镇建设，完成新区道路及绿化亮化工程，加快公共服务设施、新区保障房建设和建成区改造，适时启动示范镇管理体制改革。制定出台倾斜支持政策，启动建设市级重点镇10个。

四是加强城市精细化管理。进一步完善城市管理体制机制，形成城管牵头、部门联动、街道办和社区负责、市民参与的“大城管”格局。启动数字化城市管理平台和“数字城市”地理空间框架建设，加强市政和环卫设施建设，组织开展乱占乱建专项整治，坚决纠正占道经营、乱停乱放、乱倒乱排行为。启动国家园林城市创建工作，创建省级园林单位2个、市级10个，完成丹凤、山阳两县省级园林县城创建任务，启动山阳创建省级卫生县城工作。

五是继续推进农村居民进城落户。进一步完善各项保障措施，大力推行一元化户籍管理，优先解决在城市出生的非城镇户籍青少年落户，今年力争实现农村居民进城落户6万人，使他们尽快融入城市生活。

(六)深入推进改革开放，加快发展开放型经济

坚定不移地推进改革，进一步扩大对外开放，创造优良发展环境，不断增强经济社会发展活力。

一是继续推进重点领域改革。稳步推进事业单位分类改革和行政事业单位国有资产管理体制改革。继续抓好财税体制改革，制定出台支持发展飞地经济的具体政策。进一步深化文化体制改革，完成县区文化事业单位改革任务。深化医药卫生体制改革，认真落实国家基本药物制度，全面推进县级公立医院改革，鼓励引导社会资本办医，加快形成多元化办医格局。深入推进国有企业改革，盘活现有存量资源。完善集体林权制度配套改革，积极推进粮食流通体制改革。

二是大力发展开放型经济。坚持“走出去”、“引进来”，用足用好外贸扶持政策，重点支持太阳能电池、有色金属和农副产品等出口，促进外贸稳步增长。以承接东部产业转移为重点，大力开展依商招商和产业链招商，力争招商引资到位资金180亿元。

三是加快发展非公有制经济。认真落实促进非公有制经济健康发展的政策措施，坚持非禁即入、降低门槛，以创新型科技企业和小型微型企业为重点，放开搞活非公有制经济。积极搭建创业辅导和咨询服务平台，强化劳动技能和市场知识培训，在释放创业潜能中增加企业数量。鼓励引导在外务工、经商成功人士以及高校毕业生回乡创业，新发展个体经营户2600家、私营企业320家，非公有制经济增加值增长20%以上。

四是进一步改善发展环境。大力营造公平透明的政策环境，进一步减少和规范行政审批，搭建各类要素交易平台，使各类企业平等享受公共资源、平等参与市场竞争。大力营造公正有序的法制环境，规范行政事业性收费，坚决制止吃拿卡要等现象。大力营造优质高效的服务环境，重视配套设施和服务体系建设，为企业发展创造良好条件。大力营造重商亲商的社会环境，珍爱企业、关爱企业家，宣传典型、鼓励创业致富，促进各类市场主体健康成长。

(七)切实保障和改善民生，让人民群众生活得更加幸福

把保障和改善民生作为政府工作的重中之重，以更大的决心和力度改善民生，将新增财力的80%以上用于民生，今年全市财政安排民生支出52亿元。

一是扎实推进移民搬迁工程。按照避灾移民搬迁、产业支撑和小城镇建设“三位一体”的要求，加大财政资金整合力度，加快推进移民搬迁集中安置点和多层移民楼建设，引导搬迁对象下山入川进城，今年完成新建移民集中安置点200个，搬迁安置2.1万户7.8万人。

二是继续抓好保障性住房建设。今年新建保障性住房15737套，其中廉租房2500套、公租房3000套、经适房500套、限价房5000套，发放租赁补贴2500户，城市棚户区改造2000套、林业棚户区改造237户，同时完成镇办公益人员4300套保障住房建设任务。进一步强化用地和资金保障，加快建设进度，确保工程质量，坚决杜绝“豆腐渣”工程。启动建设住房保障信息系统，面向社会公开房源和保障对象，完善分配、管理和退出制度，真正把老百姓热切期盼的住房问题解决好。

三是努力保持就业形势稳定。认真落实扩大就业的各项政策，加强职业培训和公共就业服务体系建设，充分发挥市场促进就业的作用，大力发展劳动密集型产业，积极开发公益性就业岗位，重点抓好高校毕业生、复员退伍军人和就业困难人员的就业工作，今年新增城镇就业1.2万人，其中下岗失业人员再就业5000人。加大农村劳动力转移力度，转移农村劳动力58.3万人，组织劳务输出51万人次，创收35亿元。

四是推动教育事业全面发展。坚决落实教育经费占财政支出22%的要求，完善学前教育、义务教育公用经费保障机制，加快城乡公办幼儿园和义务教育学校标准化建设，新建和改扩建中小学校80所，新建城镇和农村公办幼儿园30所，市幼儿园扩建完成主体工程。加强职业教育资源整合，支持高等教育创新发展。加大教育强县创建力度，力争实现教育强县零突破。进一步实施好蛋奶工程和农村义务教育学生营养改善计划。认真落实国家各项助学政策，为每一个孩子创造享受公平教育的机会。

五是进一步完善社会保障体系。全力推动养老、医疗保险从制度全覆盖迈向人群全覆盖，解决好集体企业职工养老遗留问题和失地农民生活保障问题，落实好环卫工人和出租车司机参加养老保险政策。进一步加强农村敬老院建设，实施10个区域敬老院扩建项目，五保对象集中供养率达到65%左右。继续提高城乡低保和农村五保供养标准，农村五保供养最低限定标准由每人每年3200元提高到3800元，其中集中供养标准提高到4000元，对机构供养“三无”人员生活费从每人每月450元提高到800元，分散供养“三无”人员每人每月发给500元生活费。认真落实城乡医疗救助和残疾人、高龄老人、孤儿补贴等政策，切实维护好各类弱势群体的切身利益。

六是不断强化医疗卫生服务保障。加快市中心医院建设步伐，抓好县区公立医院和4个中心卫生院、2个城市社区卫生服务中心标准化建设。将新型农村合作医疗和城镇居民基本医疗保险人均财政补助标准提高到250元，并进一步提高报销比例。深入实施农村孕产妇免费住院分娩、65岁以上老人健康检查、农村儿童白血病和先天性心脏病救治保障、城乡居民健康档案建档、重大传染病地方病预防等公共卫生服务项目。大力实施全科医生培训计划，争取年内每个社区卫生服务机构和镇办卫生院都有合格的全科医生。积极创建人口和计划生育国家级综合改革示范市，稳定低生育水平，提高人口素质。

七是加快发展文化体育等事业。进一步完善公共文化服务体系，积极实施农村文化建设五大工程，完成镇办广播电视“村村通”建设任务，在32个城市社区建立公共电子阅览室，为100个村(社区)文化活动室配送文化活动器材。加快市体育场和体育主体公园建设，落实全民健身计划，实施农民体育健身项目40个。大力发展城市公共交通，继续做好养老服务和妇女儿童权益保障工作，加快发展社会福利、红十字会和慈善事业。

(八)加强和创新社会管理，筑牢社会和谐稳定的基础

坚持以人为本、执政为民，着力完善制度安排、解决突出问题，全面提升创新社会管理能力，创造和谐稳定的发展环境。

一是健全群众利益维护机制。坚持领导接访、下访、回访和包案等制度，严格落实信访工作责任制，大力推行联合接访机制，加强行政复议和行政调解，统筹协调各方面利益关系，强化社会矛盾源头治理，妥善处理人民内部矛盾，尤其解决好征地拆迁、土地流转等容易引发集体上访的问题，坚决纠正损害群众利益的不正之风。进一步深化信访“三无”县区和镇办创建工作，确保实现“一控三降”目标。依法管理宗教事务，保障少数民族的合法权益。

二是切实加强城乡社区建设。明确社区服务内容和工作规范，加大资金、设施、人员等方面的支持力度，同时引导各类社会组织加强自身建设，充分发挥群众自治组织在社会管理中的作用。加快市级社区服务信息网络平台和县级社区服务中心建设，改扩建城市社区服务站20个、农村社区服务站61个。

三是做好重点人群服务工作。切实关爱农村留守儿童、妇女和老人，进一步做好街头流浪乞讨人员的收容遣送工作，加强青少年维权、预防违法犯罪、刑释解教人员和社区矫正工作，强化心理疏导和人文关怀，不断完善两新组织管理措施，努力实现管理服务全覆盖。

四是加强社会诚信体系建设。广泛开展诚信宣传教育活动，健全完善诚信制度和奖惩监管机制，建立诚信信息公开共享平台，全面推进政府、企业和个人诚信建设，不断增强公民明礼诚信的自觉意识和公德意识，营造讲诚信、守信用、用信用的良好社会氛围。

五是强化社会治安综合治理。深入推进平安创建活动，加强基层政法机构建设，健全治安防控体系。积极推进科技强警和公安队伍建设，深入开展重点领域的社会治安整治，严厉打击各类刑事犯罪活动。加强应急队伍建设，建成应急指挥中心，提高应急处置能力。切实加强国家安全工作，严密防范敌对势力的渗透和破坏。支持驻商部队正规化建设，深入开展国防教育，启动建设民兵训练基地和市公安消防特警中队营房工程。

六是全面加强安全生产工作。夯实企业主体责任和政府监管责任，强化安全生产过程监管，建立事前问责机制，严格事后责任追究。大力推进重点行业、企业安全生产标准化建设，深入开展安全生产专项整治，加强城乡公共消防安全管理，及时消除各类不安全隐患，坚决遏制重特大安全事故发生。按照中省有关规定，加强校车管理，确保校车安全。建立健全食品药品安全监管机制，确保人民群众饮食用药安全。

(九)不断提升行政效能，建设为民务实清廉政府

站在新起点，促进新发展，开创新局面，对政府工作提出了新的更高要求，我们必须不断加强政府自身建设，提升履职尽职工作水平，为建设幸福商洛做出新贡献。

一是始终牢记执政为民的工作宗旨。秉承以人为本、执政为民的工作理念，重大决策顺民而定，发展举措应民而出。把群众的呼声作为政府工作的第一信号，从群众最关心的热点问题抓起，从群众最直接的生计问题抓起，从群众最现实的利益问题抓起，努力创造更多的发展成果惠及人民，让广大群众过上更加舒心而美好的生活。

二是始终保持创新奋进的工作状态。面对经济全球化日益加深和复杂多变的新形势，我们必须加强对新政策、新知识、新理论成果的学习，加强对前瞻性、战略性、规律性问题的分析，不断提升领导科学发展的能力和水平。深入推进思想解放和观念创新，强化赶超意识，奋力争先进位，把敢创与善谋融为一体，将实干与巧干有机结合，努力在竞相发展的浪潮中抢占先机。

三是始终践行求真务实的工作作风。深入推进机关干部“治庸、治懒、治散”，大力倡导“先干不议论、快干不争论、实干不空论、发展做结论”的理念，转变工作作风，改进文风会风，减少事务应酬，做到服务发展不遗余力，服务群众尽心尽力。进一步完善抓落实工作机制，继续加大目标考核、问责问效力度，严格执行首问负责、限时办结和过错追究等制度，努力增强政府的执行力和公信力。

四是始终坚持依法行政的工作规范。认真实施法治政府建设五年规划，扎实推进“六五”普法工作，实现省级依法行政示范市创建目标。更加自觉地接受市人大的法律监督，更加自觉地接受市政协的民主监督，认真办理人大代表议案、建议和政协委员提案，主动听取各民主党派、工商联、无党派人士和各人民团体的意见。健全新闻发布和信息公开制度，大力推进政务公开，逐步推进网络问政，不断提高政府工作的透明度。

五是始终秉持清正廉洁的工作操守。以敬畏之心对待权力，带头遵守廉洁从政各项规定，筑牢拒腐防变的思想防线，坚守清正廉洁的人生底线，秉公用权，干净做事，清白做人。以坦诚之心对待监督，认真落实述职述廉、重大事项报告、经济责任审计等制度，主动接受多种形式和途径的监督，使监督成为干好工作的重要保证。以宽容之心对待同志，倍加珍惜和维护班子团结，用坚强的党性保证团结，用高尚的人格增进团结，用共同的事业维护团结，不断提高政府班子的凝聚力和战斗力。

各位代表，新的蓝图宏伟而壮丽，新的使命崇高而光荣。让我们高举中国特色社会主义伟大旗帜，深入贯彻落实科学发展观，在市委的坚强领导下，团结带领全市人民，开拓创新，锐意进取，同心同德，顽强拼搏，以优异成绩迎接党的十八大和省第十二次党代会胜利召开！
